# The Interactive Effect of EFL Teachers’ Emotions and Cognitions on Their Pedagogical Practices

**DOI:** 10.3389/fpsyg.2021.811721

**Published:** 2021-12-06

**Authors:** Yan Shi

**Affiliations:** School of Languages and Media, Anhui University of Finance and Economics, Bengbu, China

**Keywords:** positive psychology, teacher emotion, teacher cognition, pedagogical practice, EFL teacher

## Abstract

Emotion and cognition have long been considered as two influential factors determining the quality of teaching and learning. They form the foundation of all aspects of teaching as an emotional and thought-provoking profession. With the advent of Positive Psychology (PP) and affective pedagogy, now English as a Foreign Language (EFL) teachers’ inner states and emotions are placed at the center of every educational program all around the world. This consideration has led to a rise in various domains of teaching and teacher education. However, the interactive influence and association between teachers’ emotions and cognitions concerning their pedagogical practices has been mostly left ignored in EFL contexts. To fill this gap, the present study aimed to offer a mini-review of the concepts, definitions, related theories, and empirical evidence in this line of research. It also presented practical implications for different stakeholders together with research gaps and future directions for enthusiastic L2 investigators.

## Introduction

For a long time, teaching was viewed as a cognitive process without taking into consideration the psycho-emotional factors involving in this complex process (Agudo, 2018; Sikma, 2021). However, with the emergence of Positive Psychology (PP), Emotionology, and the Affective Turn in pedagogy, emotions and inner feelings were given a pivotal role in education (Gregersen and MacIntyre, 2021). This paradigm shift was a movement against the traditional static, essentialist, and experimental perspectives which left no room for one’s inner states and emotions in academia (Bigelow, 2019; Kırmızı and Sariçoban, 2020). Teaching as a social practice involves various factors and elements which have made it as one of the most demanding professions in the world (Benevene et al., 2020). Due to their exponential and additive impacts, emotions play an outstanding role in language education as teachers and leaners are grappling with a language other than their own native language. This, in turn, multiplies the emotional tensions and pressures on EFL/ESL teachers to stay positive and bring about desirable academic outcomes (King and Ng, 2018). Now that emotions have firmly positioned themselves in the body of knowledge in applied linguistics and social sciences, teachers’ emotional factors and their positive and negative influences on various educational aspects have been dragged to light from the shadow of cognitive views. As a result, a growing body of research from different parts of the work have been carried out to unravel the mystery of different teacher emotions and their associated outcomes (e.g., Derakhshan et al., 2019; Greenier et al., 2021; Li and Yang, 2021; Pishghadam et al., 2021; Xie and Derakhshan, 2021; Zhao and Li, 2021). As research indicates, teachers’ emotions in second/foreign language education have a penetrating effect on numerous variables such as their motivation, engagement, efficacy, interpersonal communication skills, resilience, academic buoyancy, professional identity (re)construction, pedagogical practices, classroom behaviors, well-being, enjoyment, effectiveness, job commitment and many more.

Additionally, teachers’ emotions or hidden internal feelings have a direct correlation with their cognition, especially their attention, thinking, mood, perception, and beliefs (Sugesti et al., 2020; Alzaanin, 2021). Teacher cognition highlights the active role of one’s thoughts and mental life on his/her actions (Borg, 2009). In L2 education, what a teacher does, knows, and believes definitely influences the teaching and learning cycle (Ghasemi, 2020). It is now unwarranted to see teaching as a process-product model that works in a linear fashion (Borg, 2006). Instead, it is a complicated profession which calls into attention the impact of emotions, thoughts, knowledge, judgments, reasoning, and beliefs on teachers’ instructional practices. All these factors form a mental system for the teachers which is influenced by personal and contextual factors including schooling and professional coursework (Borg, 2006). As pinpointed by Ghasemi (2020), teacher cognition is able to affect various classroom behaviors and actions. When a teacher has organized mental patterns, sufficient knowledge, positive attitudes and beliefs, and knows what to do in a classroom, he/she takes professional steps in teaching and generates optimal outcomes. Although research certifies the effect of teacher cognition on identity development, teaching different skills, assessment, materials selection/use, decision-making, and reflection, the go togetherness of teacher emotion and cognition has remained a mystery in EFL contexts. To fill this research gap, the present mini-review article aims to present the definitions, concepts, applications, and possible lines of research concerning EFL teachers’ emotions, cognitions, and their pedagogical practices.

## Theoretical Background

### Emotion in Education: Origins and Impacts

Like other life-domains, human’s emotions and internal feelings are at the core of success in education as well. People carry with themselves their own feelings wherever they go as they are emotional creatures. The power of psycho-emotional factors in human being’s is so remarkable in the sense that it permeates into every aspect of one’s career and performance. However, for decades teaching and learning were only seen as cognitive processes and scholars largely turned a blind eye on the social-emotional essence of human behavior and practice (Li, 2021). The mystery remains unraveled until humanistic psychology and its related extensions emerged in psychology and education. Such trends placed a pivotal emphasis on the effect of emotions in education and language teaching/learning. Breakthroughs in teacher-psychology variables happened owing to the rise of positive psychology (PP), emotionlology, and affective pedagogy. These trends accentuated the role of emotions in development, success, and happiness (Seligman, 2011; Pavlenko, 2013; Alba-Juez and Mackenzie, 2019; MacIntyre et al., 2019; Zhang and Zhang, 2020).

Now in language education and teacher education, the focus has shifted from negative emotions to positive sides of teachers’ career to incur desired outcomes. This paved the way for the conduction of several studies on teacher emotions such as efficacy, self-esteem, motivation, engagement, love, interpersonal abilities, resilience, praise, immediacy, stroke, enjoyment, and hope which were identified to influence teachers’ classroom practices (e.g., Dewaele et al., 2019; Fathi et al., 2020; Zhang and Zhang, 2020; Pishghadam et al., 2021; Wang et al., 2021; Xie and Derakhshan, 2021). These studies remarkably substantiate that teaching is a highly emotion-oriented job and teachers are the cores of such a system.

### The Concept of (Teacher) Emotion

The notion of emotion is as broad as the number of researchers who attempted to define it. It is a complex construct which has been defined differently by educators. According to Nyklíček et al. (2011), emotion is a phenomenon related to one’s functioning and value that improves effectiveness and helps in accomplishing pre-specified goals. Furthermore, emotion is regarded as dynamic, multi-layered construct which is psycho-social in nature and is a momentary feeling that causes adaptability to a sensitive situation (Pekrun, 2006; Mercer, 2020). It is a mental state which has explicit causes and outcomes. Depending on the duration and situation, emotions can be state or trait. Trait emotions are frequent reactions and tendencies to an encounter, while state emotions are short-lived and ephemeral reactions to a specific situation or event. Although the root of emotion is inside the individual, it does not remain inert to the body and represents itself in one’s behaviors and interactions in social contexts. Hence, it can be argued that teachers’ emotions are shaped by and shaping internal and external aspects of language education. In the pertinent literature, there are other cognate terms related to concept of emotion which have sometimes been used interchangeably. They include mood, affect, feeling, cognition. *Mood* is a moderately constant, self-regulated affective state occurring without any specific reason/cause (Bryan et al., 1996; Newton, 2013). *Affect* is a broad concept which covers both emotion and mood. It is one’s basic feeling regardless of being pleasant or unpleasant. Moreover, *feeling* is one’s sensory perception of something which is a private manifestation of emotion experienced by the person. It does not have physiological and behavioral element in contrast to emotion (Ghasemi, 2020). Finally, emotion differs from *cognition* or mental thoughts in that cognition does not have facial expression and the physiological element of emotion. Despite these differences, they can work together toward an identical aim.

### Teacher Cognition

Teacher cognition is a theoretical construct/framework which specifies the complexity of teachers’ mental lives (Borg, 2006). It can be defined as the hidden cognitive dimension of teaching or what teachers know, believe, and think (Borg, 2003). This complicated variable emanates from teachers’ experiences, personalized and contextualized outlooks, and attitudes (Farrell and Lim, 2005). As stated by Borg (2006), teacher cognition is a concept related to their thoughts, knowledge, and beliefs which influences their educational decisions and practices. Going even further, language teachers’ cognition refers to teachers’ system of beliefs, knowledge, theories, attitudes, assumptions, conceptions, principals, thinking, and decision-making about teaching, teachers, learners, learning, subject matter, curricula, materials, activities, self, colleagues, assessments, and context (Borg, 2006). According to this definition, teachers’ cognition covers and pertains to almost all aspects of their job. This overarching construct can also be considered as a field of research and practice whose dynamics is shaped by four factors including teachers’ language learning experiences, job, contextual issues, and classroom practices.

### Teacher Cognition and Classroom Practices

Undoubtedly, teachers’ cognition and mental processes determine each and every moment of their instruction. As cognition encompasses different teacher-related variables and aspects of teaching, it is not weird to expect that EFL teachers who have sufficient knowledge and awareness of their mental life perform better in the class and use more effective strategies. Teachers’ pedagogical practices are not confined to their methodology and techniques, but they cover various steps that they take to sound like a professional teacher who is able to guide and direct students toward their ultimate purpose (i.e., learning). Tracing back the related studies in this research territory, one can come across different studies on the multifarious effect of teachers’ cognition on different pedagogical practices (Başar, 2020). Research demonstrates that teacher cognition which functions like a cognitive filter or an umbrella that covers all aspects of a teacher’s profession has the potentiality to affect teachers’ perception and practice of corrective feedback, different language skills and sub-skills, professional development trajectories, classroom methodologies, and assessment techniques (e.g., Chappell et al., 2015; Sato and Oyanedel, 2019; Başar, 2020; Moradkhani and Goodarzi, 2020; Sugesti et al., 2020; Sun et al., 2020).

Drawing on the definition of teacher cognition proposed by Borg (2006), one can claim that cognition in EFL contexts can influence teachers’ beliefs, values, identity as an EFL teacher, perceptions of specific instructional strategies, materials development, classroom tasks, practical knowledge, professional reasoning, discursive interactions, and interpersonal relationships and rapports among teachers, students, colleagues, and parents. These propositions make it clear that teacher cognition has to do with all academic domains.

### The Interconnection of Teacher Emotion and Cognition

From the time of Socrates, cognition and emotion have been considered as two separated and mutually exclusive worlds. This was due to the prominence and emphasis placed on though and reasoning over one’s emotion and affect (Swain, 2013). Against this perspective, Vygotsky’s socio-cultural theory (SCT) of mind approved the interrelationship between one’s cognition and emotion. This was done through the notion of *perezhivanie* which is defined as the emotional experience emerging from any situation or context (Vygotsky, 1994). It is a lived emotional experience that leaves an enduring effect on an individual and somehow changes his/her development (Blunden, 2016). Based on SCT, experiencing something occurs in the sphere of emotion and its processing occurs in the sphere of cognition. That is why Vygotsky regarded these two concepts as intertwined. Although teaching has been approved to be all about “being and feeling” and many studies grounded in PP highlighted the role of the emotional dimensions of teaching both in mainstream education and language education, the relationship between EFL teachers’ emotion and cognition has not been methodically explored, to date (Borg, 2003; Zembylas, 2007). There is a dire need for empirical research on these critical factors related to teacher psychology in order to provide a vivid image of these concepts and their interactive effects in language education.

### Implications, Research Gaps, and Future Trends

In this mini-review article, it was argued that EFL teachers’ emotional factors influence different aspects of their career. Moreover, it was claimed that emotion and cognition are two intertwined constructs which are able to affect various teaching and testing practices of EFL teachers. Hence, the study can be of value for EFL teachers in that their knowledge, awareness, and practice of psych-emotional aspects of language teaching can exponentially increases. They also realize that their mental beliefs and perceptions influences their pedagogical practices, to a large extent.

Teacher educators are the second camp which can benefit from the ideas of this mini-review in the sense that they can plan and offer effective training programs for EFL teachers to raise their knowledge of emotional aspects of teaching aside from pedagogical issues. They can inform EFL teachers that teaching is not only about methodology and techniques to teach something, but a complex job including numerous emotional and mental elements that are essential to be called upon when teaching a class.

Moreover, materials developers can use this study to write appropriate textbooks and activities in which EFL teachers’ emotions and cognition are reflected and tapped. Boring textbooks and materials can hamper both emotion and pedagogy. Therefore, based on this research, new materials are required for EFL teachers as the current ones lack the element of emotion in their contents. Finally, L2 researchers might find this study valuable in that they can make attempts to bridge the existing gaps in this line of research.

First, empirical research on the correlation among EFL teacher/emotion, cognition, and pedagogical practice is scant. Therefore, future studies are recommended to interested researchers. Second, the dynamism of emotional and mental variables in L2 education requires longitudinal and qualitative research instruments by future scholars. Additionally, cross-cultural studies can be done on these variable to identify if contextual factors make a significant change or not. Likewise, future studies can be conducted to study other positive emotion variables proposed by PP (see Xie and Derakhshan, 2021) in relation to cognition. Last but not least, the effect of teacher cognition of interpersonal communication skills such as credibility, clarity, immediacy, and confirmation is also an exciting direction for future explorations.

## Author Contributions

The author confirms being the sole contributor of this work and has approved it for publication.

## Conflict of Interest

The author declares that the research was conducted in the absence of any commercial or financial relationships that could be construed as a potential conflict of interest.

## Publisher’s Note

All claims expressed in this article are solely those of the authors and do not necessarily represent those of their affiliated organizations, or those of the publisher, the editors and the reviewers. Any product that may be evaluated in this article, or claim that may be made by its manufacturer, is not guaranteed or endorsed by the publisher.
